# High-electron-mobility (370 cm^2^/Vs) polycrystalline Ge on an insulator formed by As-doped solid-phase crystallization

**DOI:** 10.1038/s41598-019-53084-7

**Published:** 2019-11-12

**Authors:** M. Saito, K. Moto, T. Nishida, T. Suemasu, K. Toko

**Affiliations:** 1 Institute of Applied Physics, University of Tsukuba, 1-1-1 Tennodai, Tsukuba, Ibaraki, 305-8573 Japan; 2 PRESTO, Japan Science and Technology Agency, 4-1-8 Honcho, Kawaguchi, Saitama, 332-0012 Japan

**Keywords:** Semiconductors, Electrical and electronic engineering

## Abstract

High-electron-mobility polycrystalline Ge (poly-Ge) thin films are difficult to form because of their poor crystallinity, defect-induced acceptors and low solid solubility of n-type dopants. Here, we found that As doping into amorphous Ge significantly influenced the subsequent solid-phase crystallization. Although excessive As doping degraded the crystallinity of the poly-Ge, the appropriate amount of As (~10^20^ cm^−3^) promoted lateral growth and increased the Ge grain size to approximately 20 μm at a growth temperature of 375 °C. Moreover, neutral As atoms in poly-Ge reduced the trap-state density and energy barrier height of the grain boundaries. These properties reduced grain boundary scattering and allowed for an electron mobility of 370 cm^2^/Vs at an electron concentration of 5 × 10^18^ cm^−3^ after post annealing at 500 °C. The electron mobility further exceeds that of any other n-type poly-Ge layers and even that of single-crystal Si wafers with *n* ≥ 10^18^ cm^−3^. The low-temperature synthesis of high-mobility Ge on insulators will provide a pathway for the monolithic integration of high-performance Ge-CMOS onto Si-LSIs and flat-panel displays.

## Introduction

Ge is a unique and attractive material as it has a higher carrier mobility than Si for both electrons and holes and is compatible with conventional Si processing^1–6^. Therefore, Ge complementary metal-oxide-semiconductor (CMOS) is promising for scaling beyond the Si-CMOS limit. In the last decade, numerical efforts on the gate stack^4–8^ and Ge-on-insulator (GOI) technologies^9–16^ have made Ge MOS field-effect transistors (MOSFETs) superior to Si-MOSFETs for both p and n channels. Considering that the most promising use of Ge-CMOS is to integrate it into Si large-scale integrated circuits (LSIs) or flat-panel displays, the GOI should be formed at low temperature in a simple process. However, single-crystal GOI technology such as, mechanical transfer^1,10^, oxidation-induced condensation^5,11^ and rapid-melting growth^12–15^ requires a single-crystal wafer or high temperature process (>900 °C).

Polycrystalline Ge (poly-Ge) thin films have been formed on insulators at low temperatures using solid-phase crystallization (SPC)^16–20^, laser annealing^21–24^, chemical vapor deposition^25,26^, lamp annealing^27,28^, seed layer technique^29^ and metal-induced crystallization (MIC)^30–34^. The poly-Ge layers are naturally highly p-type because of their defect-induced acceptors^35^. Although the low solid solubility of n-type dopants in Ge made it difficult to produce n-type poly-Ge in a low thermal budget^36,37^, some techniques including short-time annealing enabled n-type poly-Ge^18,24,38^. However, poly-Ge with high carrier mobility (>200 cm^2^/Vs) was difficult for both p- and n-type because of grain boundary scattering or metal contamination. Therefore, the poly-Ge MOSFETs performed worse than single-crystal GOI-MOSFETs.

We achieved a Hall hole mobility of over 340 cm^2^/Vs using SPC^39^, which has many advantages over other methods, including no metal contamination, no melting-induced surface-ripples and a simple process. Using the SPC-Ge layer on a glass substrate, we demonstrated the best transistor operation among poly-Ge-MOSFETs without minimizing the channel region^40^. The formation of GeO_2_ underlayer further improved the Hall hole mobility of poly-Ge to 620 cm^2^/Vs^41^, which greatly exceeds that of bulk-Si (430 cm^2^/Vs). We also achieved n-type poly-Ge by the SPC of Sb-doped amorphous Ge (a-Ge)^42^. However, the electron mobility was limited to 210 cm^2^/Vs by the neutral Sb scattering because of the low solid solubility of Sb in Ge. In this study, we examined As as a dopant because it has a higher solid solubility in Ge than Sb^36,37^. The As doping in a-Ge significantly changed the subsequent SPC characteristics, including crystallization rate, grain size and electrical properties. By controlling the As amount and SPC conditions, the highest electron mobility among n-type poly-Ge is achieved.

## Experimental

**Figure 1:**
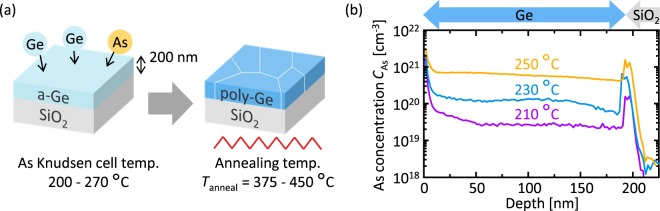
Experimental procedure of SPC of As-doped a-Ge layers. (**a**) Schematic of the sample preparation. (**b**) Representative SIMS depth profiles for the samples with an As Knudsen cell temperature of 210, 230, and 250 °C.

As schematically shown in Fig. 1(a), As-doped a-Ge layers were deposited on SiO_2_ glass substrates at RT using a Knudsen cell of a molecular beam deposition system (base pressure: 1 × 10^−7^ Pa). The Ge thickness was 200 nm and the Ge deposition rate was fixed at 1.7 nm/min. The temperature of the As Knudsen cell ranged from 200 to 270 °C to modulate the As concentration *C*_As_ in Ge. As representatively shown in Fig. 1(b), secondary ion mass spectrometry (SIMS) identified the As concentrations as 1.0 × 10^19^, 2.8 × 10^19^, 6.2 × 10^19^, 1.2 × 10^20^, 2.8 × 10^20^, 5.9 × 10^20^, 1.0 × 10^21^ and 1.8 × 10^21^ cm^−3^ at a depth of 100 nm when the As Knudsen cell temperature was 200, 210, 220, 230, 240, 250, 260 and 270 °C, respectively. The samples were then loaded into a conventional tube furnace under a N_2_ (99.9%) atmosphere and annealed at a temperature *T*_anneal_ of 375–450 °C to induce SPC. We performed post annealing (PA) at 500 °C for 5 h on all samples. According to the SIMS measurements, *C*_As_ remained constant before and after annealing.

## Results and Discussion

**Figure 2:**
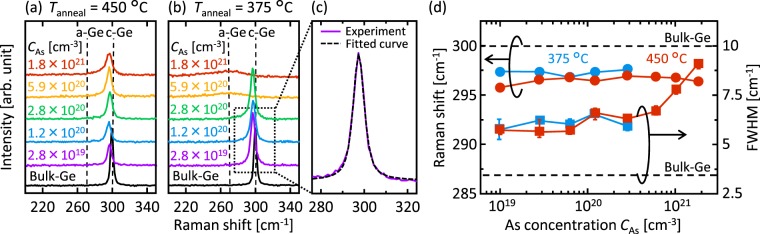
Raman spectroscopy study of the As-doped SPC-Ge layers. (**a**,**b**) Raman spectra of the samples with *C*_As_ = 2.8 × 10^19^, 1.2 × 10^20^, 2.8 × 10^20^, 5.9 × 10^20^ and 1.8 × 10^21^ cm^−3^ annealed at (**a**) 450 °C for 5 h and (**b**) 375 °C for 150 h. The spectra for a bulk-Ge wafer are shown for comparison. (**c**) Fitting result of a Raman spectrum. (**d**) Raman shifts and FWHMs of the c-Ge peaks for samples with *T*_anneal_ = 375 °C and 450 °C as a function of *C*_As_, where the values were averaged over three measurements for each sample. The data for a bulk-Ge wafer are shown by the dotted lines.

We examined the *C*_As_ dependence of the crystal quality of Ge using Raman spectroscopy (JASCO NRS-5100, spot diameter 20 μm, wavelength 532 nm). The samples with *T*_anneal_ = 450 °C exhibit sharp peaks near 300 cm^−1^, which correspond to crystalline (c-) Ge-Ge bonding in the whole *C*_As_ range (Fig. 2(a)). As shown in Fig. 2(b), annealing at 375 °C for 150 h crystallized the samples with *C*_As_ ≤ 2.8 × 10^20^ cm^−3^, but not those with *C*_As_ > 2.8 × 10^20^ cm^−3^. These results mean that excessive As lowers the crystallization rate. To analyze the Raman shift and the full width at half maximum (FWHM) of crystalline Ge (c-Ge) peaks, each spectrum was fitted as representatively shown in Fig. 2(c). The peak is fitted well enough to correctly calculate the FWHM and peak position. The Raman shift and FWHM results are summarized in Fig. 2(d). All peaks shifted to lower wavenumbers than that of a single-crystal bulk-Ge substrate, originating from the tensile strain. The peak shifts are almost constant with respect to *C*_As_ while the peaks for *T*_anneal_ = 450 °C shifted to the lower wavenumber than that for *T*_anneal_ = 375 °C. The Raman shift had small variation (<0.5%), and therefore, seems to be the dominant difference with respect to the annealing temperature. This behavior suggests that the strain likely originates from the difference in the thermal expansion coefficients between Ge and the SiO_2_ substrate. The FWHM is almost constant for *C*_As_ ≤ 5.9 × 10^20^ cm^−3^ and significantly increases for *C*_As_ > 5.9 × 10^20^ cm^−3^. This indicates that excessive As negatively influences SPC-Ge crystallinity, as will become clear in the later-mentioned electron backscattering diffraction analyses. Thus, the Raman studies revealed that *C*_As_ strongly influences the growth rate and crystal quality of SPC-Ge.

**Figure 3:**
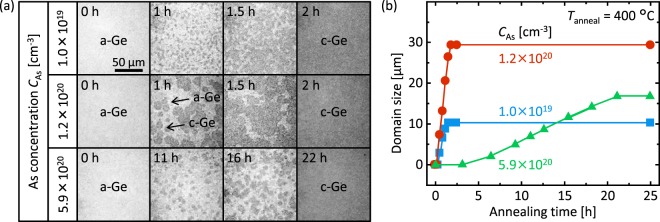
Characteristics of the growth rate of As-doped (*C*_As_ = 1.0 × 10^19^, 1.2 × 10^20^ and 5.9 × 10^20^ cm^−3^) SPC-Ge. (**a**) *In situ* optical microscopy observation. The light-colored area indicates a-Ge and the dark-colored area indicates c-Ge. (**b**) Annealing time dependence of the domain size derived from a typical domain in the micrographs for each *C*_As_. Here *T*_anneal_ = 400 °C.

The *C*_As_ dependence of the growth rate was evaluated using *in situ* optical microscopy during annealing. Figure 3(a) shows the typical growth evolution of SPC. Here we chose *T*_anneal_ = 400 °C because it allowed for both domain visibility and practical observation time in a wide range of *C*_As_. The micrographs indicate that Ge nucleation occurs and the domain grows laterally with increasing annealing time. Eventually, the entire surface is covered with c-Ge for each sample, indicating that the SPC (lateral growth of domains) is saturated. The domain growth rate and saturated domain size vary significantly with *C*_As_ (Fig. 3(b)). The medium *C*_As_ sample (*C*_As_ = 1.2 × 10^20^ cm^−3^) exhibited the highest growth rate and the largest domain size among the three samples. Generally, impurity doping promotes semiconductor atom migration and enhances the recrystallization rate of amorphous films^43^. Conversely, excessive As reduces both nucleation and lateral growth rates (Fig. 3(b)). This is likely because segregation of excessive As suppressed nucleation and growth. These behaviors have also been reported in Sn- and Sb-doped SPC-Ge^42,44,45^. Therefore, As doping in a-Ge greatly influences nucleation and lateral growth in subsequent SPC.

**Figure 4:**
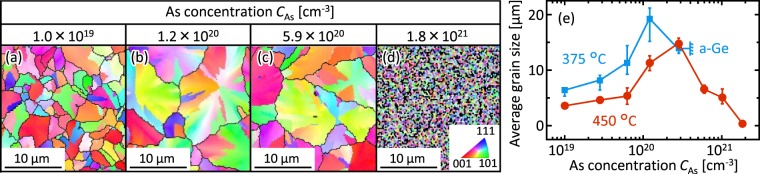
Grain size of the As-doped SPC-Ge layers. (**a**–**d**) IPF images of the samples annealed at 450 °C with *C*_As_ = (**a**) 1.0 × 10^19^, (**b**) 1.2 × 10^20^, (**c**) 5.9 × 10^20^ and (**d**) 1.8 × 10^21^ cm^−3^. (**e**) Average grain size determined by the IPF analyses for samples with *T*_anneal_ = 375 °C and 450 °C as a function of *C*_As_. For each sample, three IPF images were taken and averaged.

The inverse pole figures (IPFs) with grain boundaries in Ge were obtained using electron backscattering diffraction analyses (JEOL JSM-7001F with the TSL OIM analysis attachment). The grain size dramatically varies with *C*_As_ (Fig. 4(a–d)). The average grain size increases with increasing *C*_As_ and then begins to decrease (Fig. 4(e)). This behavior agrees with that of the eventual domain size in optical micrographs (Fig. 3). Additionally, the grain size is significantly degraded by excessive As (*C*_As_ = 1.8 × 10^21^ cm^−3^). This behavior well accounts for the results of the Raman FWHM (Fig. 2(d)). The lower *T*_g_ provides a larger grain size, which agrees with the general tendency of SPC-Ge reflecting the reduction of nucleation frequency^17,39^. The sample with *C*_As_ = 1.2 × 10^20^ cm^−3^ and *T*_g_ = 375 °C exhibited a grain size of approximately 20 μm, which is the largest among poly-Ge formed by SPC.

**Figure 5:**
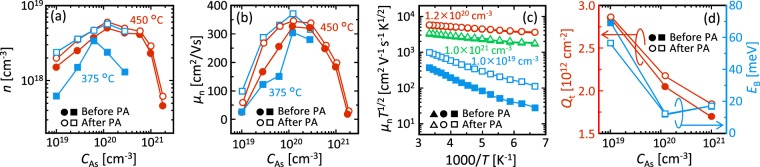
Electrical properties of the As-doped SPC-Ge layers. (**a**) Electron concentration *n* and (**b**) electron mobility *μ*_n_ for *T*_anneal_ = 375 °C and 450 °C before and after PA (500 °C) as a function of *C*_As_. (**c**) Arrhenius plot of *µ*_n_*T*^1/2^ for samples with *C*_As_ = 1.0 × 10^19^, 1.2 × 10^20^ and 1.0 × 10^21^ cm^−3^ for *T*_anneal_ = 450 °C before and after PA. (**d**) Trap-state density *Q*_t_ and energy barrier height *E*_B_ of the Ge grain boundary as a function of *C*_As_. Here *n* and *μ*_n_ were averaged over five measurements for each sample, where the variation was smaller than the marks.

The electrical properties of the As-doped SPC-Ge layers were evaluated using Hall-effect measurements with the van der Pauw method (Bio-Rad HL5500PC). All samples showed n-type conduction owing to the self-organizing activation of As during SPC. Electron concentration *n* and electron mobility *μ*_n_ depend on both *T*_anneal_ and *C*_As_ (Fig. 5(a,b)). We note that the maximum variation between samples prepared under the same conditions is approximately 20% in *n* and 5% in *μ*_n_, while the measurement variation was smaller than the marks in the figures for each sample. We first discuss the *T*_anneal_ dependence of the electrical properties. Before PA, *n* for *T*_anneal_ = 450 °C is higher than that for *T*_g_ = 375 °C in the whole *C*_As_ range (Fig. 5(a)). This behavior is consistent with the fact that higher temperatures cause higher solid solubility and activation of As in Ge^36^. *T*_anneal_ = 450 °C exhibits a higher *μ*_n_ than *T*_anneal_ = 375 °C (Fig. 5(b)), whereas the grain size shows the opposite trend (Fig. 4(e)). According to the carrier conduction model proposed by Seto for polycrystalline semiconductors^46^, the energy barrier of the grain boundary *E*_B_ decreases as the carrier density increases. The *T*_anneal_ dependence of *μ*_n_ is likely attributed to the fact that *T*_anneal_ = 450 °C has higher *n* and therefore lower *E*_B_ than *T*_anneal_ = 375 °C. After PA at 500 °C, *n* for *T*_anneal_ = 450 and 375 °C increases to a similar value for each *C*_As_ (Fig. 5(a)). These results suggest that the activation rate of As in Ge is determined by the maximum process temperature. *μ*_n_ is improved by PA for both *T*_anneal_ (Fig. 5(b)). In particular, *μ*_n_ for *T*_anneal_ = 375 °C is higher than that of *T*_anneal_ = 450 °C, which reflects the grain size (Fig. 4(e)). After PA, both *n* and *μ*_n_ are maximized at around *C*_As_ = 1.2 × 10^20^ cm^−3^ where the grain size is maximum (Fig. 4(e)). The *C*_As_ dependence of *n* is likely because the larger grain size provides the lower defect-induced acceptors and/or the less As segregation at grain boundaries. Although the *C*_As_ dependence of *μ*_n_ is consistent with the tendency of grain size, the dramatic improvement of *μ*_n_ from *C*_As_ = 1.0 × 10^19^ cm^−3^ to *C*_As_ = 1.2 × 10^20^ cm^−3^ is difficult to explain only in terms of grain size.

**Figure 6:**
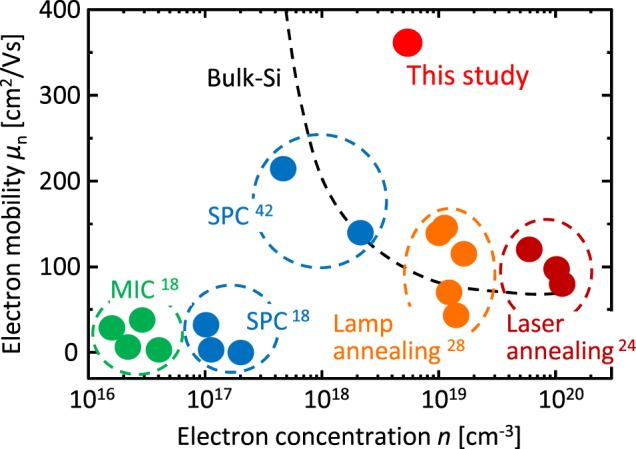
Comparison of the electron mobility *µ*_n_ and electron concentration *n* of n-type poly-Ge layers formed on insulators. The growth method and the reference number are shown near each symbol. The data for single-crystal bulk Si is shown by the dotted line.

To clarify the behavior, we quantified the trap-state density *Q*_t_ in the grain boundaries and *E*_B_ using the following equations^46^:

1 $${\mu }_{n}{T}^{1/2}=\frac{{Lq}}{\sqrt{2{\rm{\pi }}m\ast k}}\exp \,(-\frac{{E}_{B}}{{kT}}),$$

2 $${Q}_{t}=\frac{\sqrt{8{\rm{\varepsilon }}n{E}_{B}}}{q},$$

where *T* is the absolute temperature, *L* is the grain size, *q* is the elementary charge, m^*^ is the effective mass, *k* is the Boltzmann constant and *ε* is the dielectric permittivity. The Arrhenius plot of *µ*_n_*T*^1/2^ makes an almost-downward-sloping straight line for the whole *C*_As_ region (Fig. 5(c)). *Q*_t_ decreases with increasing *C*_As_, which suggests that As atoms passivate the grain boundary traps (Fig. 5(d)). Therefore, *E*_B_ dramatically decreases by As doping at *C*_As_ = 1.2 × 10^20^ cm^−3^, which reflects both the decrease of *Q*_t_ and increase of *n*. On the other hand, *Q*_t_ slightly increases with PA. This behavior is likely because PA increases lattice substitutional As and therefore reduces the extent to which As passivates the grain boundary. After PA, *E*_B_ for *C*_As_ = 1.2 × 10^20^ cm^−3^ does not change, which reflects the balance between *Q*_t_ and *n*, while *μ*_n_ improves slightly (Fig. 5(b)). Considering that PA improves the activation rate of As, the *μ*_n_ improvement is likely due to the decrease of carrier scattering by neutral As. The *n* and *µ*_n_ values reached 5 × 10^18^ cm^−3^ and 370 cm^2^/Vs, respectively. The *µ*_n_ value further exceeds that of any other n-type poly-Ge layers formed on insulators and even that of single-crystal Si wafers with *n* ≥ 10^18^ cm^−3^ (Fig. 6).

## Conclusions

As doping into a-Ge significantly influenced the subsequent SPC. Although excessive As doping degraded the crystallinity of poly-Ge, the appropriate amount of As (~10^20^ cm^−3^) promoted the SPC and increased the Ge grain size. By combining slow annealing at low temperature (375 °C), the grain size reached approximately 20 μm, which is the largest among SPC-Ge. Moreover, neutral As atoms in Ge reduced *Q*_t_ (2 × 10^12^ cm^−2^) and *E*_B_ (12 meV). These properties reduced grain boundary scattering and allowed for *μ*_n_ of 370 cm^2^/Vs, which is the highest among n-type poly-Ge formed on insulators. These findings will provide a means for the monolithic integration of high-performance Ge-CMOS onto Si-LSIs and flat-panel displays.
